# Mycetoma in Uganda: A neglected tropical disease

**DOI:** 10.1371/journal.pntd.0008240

**Published:** 2020-04-29

**Authors:** Richard Kwizera, Felix Bongomin, David B. Meya, David W. Denning, Ahmed H. Fahal, Robert Lukande

**Affiliations:** 1 Infectious Diseases Institute, College of Health Sciences, Makerere University, Kampala, Uganda; 2 Department of Medical Microbiology & Immunology, Faculty of Medicine, Gulu University, Gulu, Uganda; 3 Department of Medicine, School of Medicine, College of Health Sciences, Makerere University, Kampala, Uganda; 4 The National Aspergillosis Centre, Wythenshawe Hospital, The University of Manchester, Manchester Academic Health Science Centre, Manchester, United Kingdom; 5 Mycetoma Research Centre, University of Khartoum, Khartoum, Sudan; 6 Department of Pathology, School of Biomedical Sciences, College of Health Sciences, Makerere University, Kampala, Uganda; Lowell General Hospital, UNITED STATES

## Abstract

Mycetoma is considered a neglected tropical disease globally. However, data on its burden and the associated complications in Uganda are limited. Hence we aimed to estimate its burden in Uganda. Firstly, a systematic PubMed search for all studies of any design on mycetoma in Uganda without restriction to the year of publication was conducted. A retrospective review of all the biopsy reports at the Pathology Reference Laboratory, Department of Pathology, Makerere University, Kampala, Uganda from January 1950 to September 2019 was conducted to identify any reports on mycetoma histological diagnosis. During the 70-years study period, 30 cases were identified by the literature review, with 249 additional cases identified by review of biopsy reports (total of 279 cases). The average incidence was estimated at 0.32/100,000 persons and prevalence of 8.32/100,000 persons per decade. However, there was a general decline in the number of cases detected recently. Males and the age group of 21–30 years were the most affected by mycetoma in Uganda, and only 7% of the cases were children. The highest number of cases was recorded from Kampala (n = 30) and Jinja (n = 19) districts. The majority of the cases (68%) were referred from surgical units. The foot was the most affected part of the body (72%). Ten per cent of the cases had bone involvement of which 58% required amputation. Fungi were the most common causative agents (89%) followed by *Nocardia* species (5%) and *Actinomycetes* (4%). The index of clinical suspicion of mycetoma was low (45%) with a very large differential diagnosis. Mycetoma is a relatively rare disease in Uganda, mostly caused by fungi, and there is a big gap in data and epidemiological studies. More systematic studies are warranted to define the true burden of mycetoma in Uganda.

## Author summary

Recently, the World Health Organisation recognized mycetoma, a chronic, progressively destructive morbid inflammatory disease usually of the foot as a neglected tropical disease. However, mycetoma is not a notifiable disease and thus the global burden of the disease remains poorly defined. It is therefore important that any collection of available data on this disease is brought forward to highlight the extent and magnitude of the burden of mycetoma. We retrospectively gathered data on the burden and aetiology of mycetoma in a country (Uganda) within the mycetoma belt based on histological diagnosis and systematic review. We found that fungal pathogens are the most common cause of mycetoma in this region. However, the distribution of the lesions is consistent with global data with the foot being the most affected site. We also noticed that clinicians have low index of clinical suspicion for mycetoma leading to under-diagnosis. Mycetoma is uncommon but not rare in Uganda; more research is obligatory to precisely define the distribution and determinants of the disease as well as primary and secondary preventative measures in endemic areas.

## Introduction

Mycetoma, originally referred to as Madura foot [1], is a progressive, chronic, granulomatous, inflammatory, subcutaneous infectious disease [2,3] caused by fungi or filamentous bacteria. The disease caused by bacteria is classified as actinomycetoma while that caused by fungi is classified as eumycetoma [4]. Mycetoma mainly affects the subcutaneous tissue, skin, muscles and may spread to involve the bone [2]. It is more common in males aged 11–40 years [5]. It has a unique geographic distribution and is known to be endemic in what is referred to as the “Mycetoma Belt”, that stretches between the latitudes of 15° South and 30° North [6]. The causative agents, which are mostly present in the soil, are introduced into the subcutaneous tissue by traumatic injury [3,6]. Hence, the foot is the most involved part of the body [5].

Late presentation for medical evaluation is common, and that is due to the painless nature of the disease in the early stages [7]. Yet, early detection and treatment are vital to reduce morbidity and improve treatment outcomes [3]. Diagnosis of mycetoma is challenging and tedious, involving many differential diagnoses which include but are not limited to foreign body granuloma, malignant tumors, tuberculosis, chromoblastomycosis and osteomyelitis [8]. However, clinical diagnosis relies on the presence of multiple nodules and numerous sinuses discharging grains [4]. Laboratory diagnosis may involve culture of the grains, histology or cytology, immunodiagnosis, polymerase chain reaction (PCR), and radiological or ultrasonic imaging [9–12]. However, PCR is the most reliable diagnostic method for ascertaining the microbial cause [2]. There is currently no serological diagnostic test for mycetoma. Actinomycetoma generally responds well to antibiotics while eumycetoma needs both long-term antifungal medication and/or surgery [6,13,14].

Mycetoma has been reported since the 1800s, but the actual global burden of the disease is unknown [3,5]. It has been reported in Asia, Latin America, Europe and Africa [2]. However, the majority of cases are reported from Sudan, India and Mexico [5]. Eumycetomas in Africa are mostly caused by *Madurella mycetomatis* [4]. There is a paucity of published data from Africa. Only about 20% of African countries have published cases of mycetoma, yet it is known to be a significant health problem in the tropical and sub-tropical regions [2,4,5]. For this reason, the World Health Organization (WHO) recently recognized mycetoma as a neglected tropical disease (NTD) during the 69^th^ World Health Assembly [15].

In Uganda, data on the burden of mycetoma and its associated complications are limited [16,17]. This study aimed to retrospectively estimate the burden of mycetoma in Uganda by reviewing biopsy reports at the Pathology reference laboratory, Makerere University, and published literature on mycetoma in Uganda.

## Methods

### Ethical statement

Ethics approval for this study was received from the School of Biomedical Sciences, Higher Degrees, Research and Ethics Committee of Makerere University, Kampala, Uganda (SBS-712).

### Study design and setting

This was a retrospective cross-sectional chart review that was carried out at the Department of Pathology, Makerere University, Kampala, Uganda, together with a systematic review of the literature to estimate the burden of mycetoma in Uganda. The department of pathology was established in 1937 to ensure the provision of medical education, research and diagnostic services at the highest standards. It makes significant contributions to the curricula of several undergraduate and postgraduate courses at Makerere University. The department engages in interdisciplinary and multi-institutional research, ranging from basic sciences, histopathology, autopsy pathology, cancer epidemiology to clinical research. It provides diagnostic pathology services to Mulago National Referral Hospital and a majority of other hospitals in Uganda in the areas of general surgical pathology, cytopathology and autopsy pathology. It is essentially “a national referral pathology laboratory” in Uganda. Uganda has six pathology laboratories under four public Universities. However, the Makerere pathology laboratory serves two National Referral hospitals, 14 Regional Referral Hospitals and two Health Centre IV facilities that are linked to the National referral hospitals.

### Literature review

A comprehensive and systematic literature search was first conducted on the burden of mycetoma in Uganda using the PubMed database without restriction to the year of publication or language. We used the following search strings; (Mycetoma*) OR Maduromycosis*) OR “Madura Foot”) OR Actinomycetoma*) OR Eumycetoma*) OR Madurella)) AND Uganda. We aimed to include all studies of any design focusing on mycetoma in Uganda, highlighting incidence, prevalence, diagnosis, treatment, mortality, distribution by age, sex, body part with lesion and causative agent. We retained the original fungal and bacterial names used in these reports, rather than adopting likely modern taxonomic synonyms.

### Review of biopsy reports

Archived books containing biopsy reports at the pathology reference laboratory, department of pathology, Makerere University, Kampala, Uganda from January 1950 to September 2019 were manually reviewed to identify any reports that had a mycetoma or Madura foot or maduromycosis as the histology diagnosis. Data were captured from these reports without patients’ identifying information and entered into Microsoft Excel spreadsheet (S1 Dataset). We only captured data on age, sex, tribe, referring unit, clinical diagnosis, histology diagnosis, body part with lesion, district of residence and causative agent. Similarly, we retained the original fungal and bacterial names used in these biopsy reports, rather than adopting likely modern taxonomic synonyms.

### Statistical analysis

Data were analyzed using STATA version 14 (STATA, College Station, Texas). The statistical analysis aimed at establishing the frequency of mycetoma over the stated period. To estimate the incidence, the number of cases diagnosed by histology per decade was divided through the mid-decade population for each decade. Population figures were derived from the World Bank (https://www.google.com/publicdata/explore?ds=d5bncppjof8f9_&met_y=sp_pop_totl&idim=country:UGA:RWA:COD&hl=en&dl=en#!ctype=l&strail=false&bcs=d&nselm=h&met_y=sp_pop_totl&scale_y=lin&ind_y=false&rdim=region&idim=country:UGA&ifdim=region&hl=en_US&dl=en&ind=false). This site only gives data from 1960 up to 2017. For the years 1950 to 1959 and 2018 to 2019, we used population figures from Worldometers (https://www.worldometers.info/world-population/uganda-population/). The current life expectancy of Uganda in 2019 is 63 years [18]. If we subtract the median age of the patients in this study (37 years), this leaves us with about 26 years, and the patient will have to live with the disease. Therefore, to estimate the period prevalence, we multiplied the incidence by 26. We analyzed the distribution of cases by sex, age and intervals of 10 years. We then described the spatial distribution by the district of residence. Results were presented as tables, graphs, maps and images as applicable.

## Results

### Literature review results

The PubMed literature search returned only two reports/citations, both of which were relevant to the topic and are briefly described below [19,20].

#### Report 1

The first citation [19] published in 1958 was a case series describing the main radiological findings of 21 cases of mycetoma among Ugandans admitted to Mulago Hospital, Kampala. In this paper, the disease was mainly confined to barefooted cultivators and more common among men than women. The average age of patients was 46 years, and only three patients complained of severe pain. The author described the discharge from the sinuses in these patients as often a blood stained serous fluid in which fungus grains were rarely found. However, it was only on histology that grains were seen but scanty. The second row of tarsals and bases of the adjoining metatarsals were the most heavily affected areas in patients with bone involvement. The main radiological characteristics of the disease were: "deep soft-tissue swelling, often with superficial nodules; irregular destruction from without, around the periphery of the affected bones (usually tarsals); sinuses within the spongiosa; a periosteal reaction which may resemble a palisade or the bristles of a brush; sclerosis of the affected spongiosa; osteoporosis distal to the affected bones and absence of true sequestra".

#### Report 2

The second citation [20] published in 1965 was a case series and review of the literature. The author compared nine cases of mycetoma diagnosed by biopsy at Mulago Hospital, Kampala over a period of 18 months in 1958/9. He then described a further study of biopsy specimens by both histological and cultural methods over a period of 14 months. Of nine specimens obtained, eight were successfully cultured: seven were *Nocardia* (6 *N*. *asteroides*), and 1 was a fungus of *Cephalosporium* species. The specimen not cultured also appeared to be a *Nocardia*. The author then analyzed the published records of mycetoma in Africa (~900 cases) on a geographical basis. *Streptomyces* infections appeared to be more common in semi-desert conditions with a rainfall of 2–10 inches per annum, *Madurella* and *Leptosphaeria* where the rainfall is between 10 and 20 inches, and *Allescheria* and *Nocardia* where it is between 20 and 80 inches.

### Results of biopsy reports reviewed

#### Incidence and prevalence of mycetoma in Uganda

**Fig 1:**
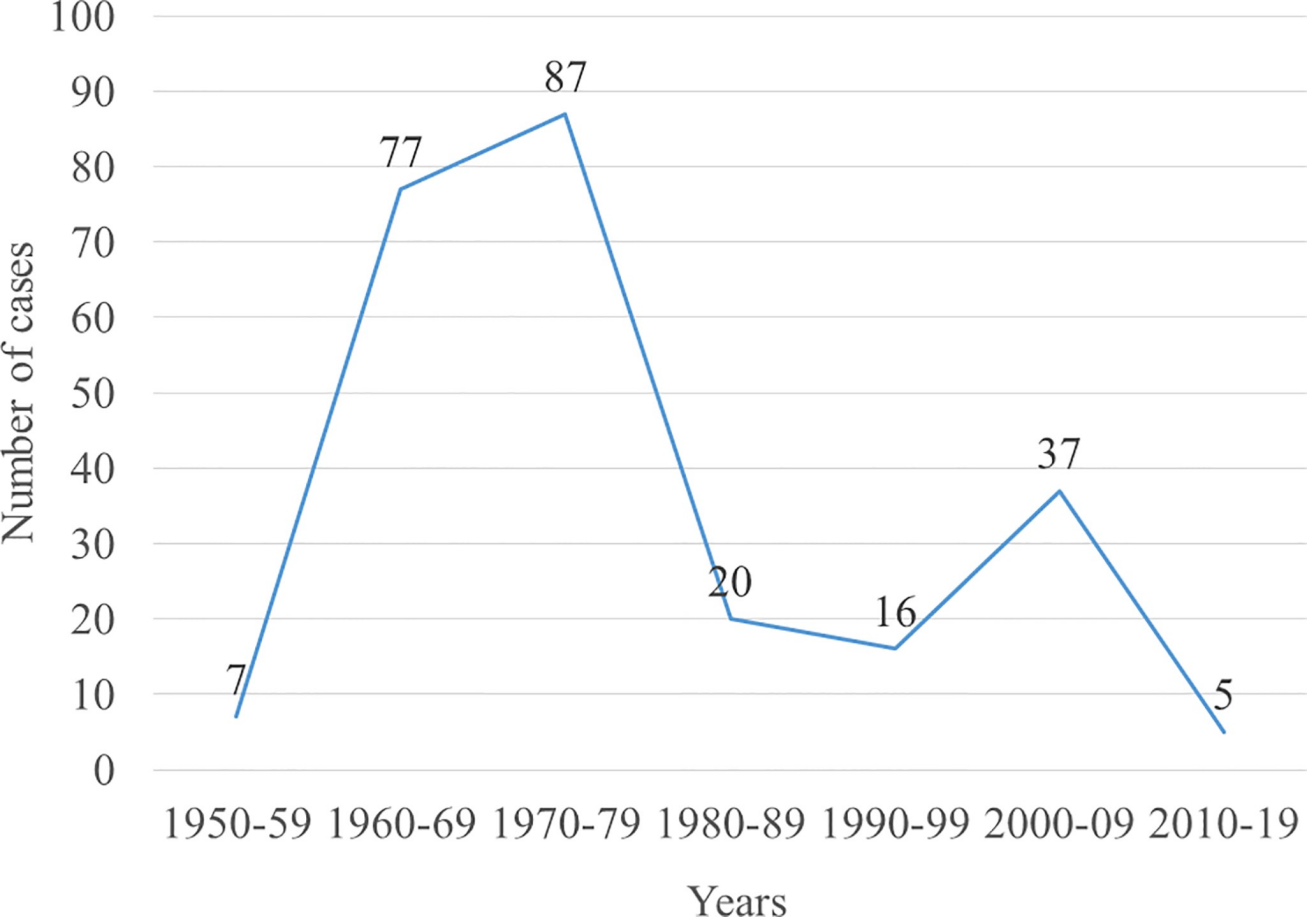
Trend in the number of mycetoma cases. There has been a gradual decrease in the number of cases identified over the years.

For the biopsy records of seventy years reviewed (1950–2019), we identified 249 cases of mycetoma diagnosed by histology (Fig 1). The number of mycetoma cases ranged from zero to 17 per year, with an average of four cases per year. The incidence per decade ranged from 0.01/100,000 to 0.96/100,000 persons with an average of 0.32/100,000 persons. Assuming that mycetoma patients live with the disease for an average of 26 years based on the Ugandan life expectancy, we estimated the prevalence per decade ranging from 0.32/100,000 to 24.98/100,000 persons with an average of 8.32/100,000 persons (Table 1). With the current population of Uganda (44,269,594), this translates into approximately 3,683 people living with mycetoma in Uganda with an estimated 142 new cases annually.

**Table 1:** Prevalence and causative agents of mycetoma in Uganda.

Years	Mid-year population	Number of biopsies done	Mycetoma cases identified	Decade Incidence per 100,000 persons	Period Prevalence per 100,000 persons	Bone involvement documented	Amputation documented	CAUSATIVE AGENTS				
								Unidentified Fungi	Actinomycetes	Nocardia species	Unidentified bacteria	Unknown cause^#^
**1950–59**	5888793	3280	7	0.12	3.09	1	1	7	0	0	0	0
**1960–69**	8014400	68407	77	0.96	24.98	11	7	64	3	10	0	0
**1970–79**	10827100	77814	87	0.80	20.89	10	7	84	1	2	0	0
**1980–89**	14646600	47473	20	0.14	3.55	0	0	16	3	0	1	0
**1990–99**	20550300	65761	16	0.08	2.02	2	0	15	0	0	1	0
**2000–09**	28543900	61079	37	0.13	3.37	1	0	32	2	0	0	3
**2010–19**	40144900	52185	5	0.01	0.32	1	0	3	1	0	1	0

**^#^**There were 3 cases whose causative agent was not mentioned in the biopsy reports.

#### Causative agents of mycetoma in Uganda

As seen in Table 1, fungi were the most common causative agents (88.8% [221/249]) followed by bacteria (10% [25/249]). There were three cases (1.2%) whose causative agent was not mentioned in the biopsy reports (Table 1). Of the 25 bacterial cases, ten were recorded as *Actinomycetes*, 12 as *Nocardia* species and three as unidentified bacteria. Periodic acid–Schiff (PAS) staining was used as a special stain to confirm fungal pathogens. However, all fungal pathogens were not identified to a genus level.

#### Index of clinical suspicion for mycetoma in Uganda

Only 113 cases (45.4%) were clinically diagnosed as mycetoma, prior to biopsy. Most cases were clinically mis-diagnosed as Kaposi sarcoma (N = 37), fungal infection (N = 2), tuberculosis (n = 4), tumor (n = 3), melanoma (n = 3), sarcoma (n = 2), fibroma (n = 2), abscess (n = 1), buruli ulcer (n = 1), fibrolipoma (n = 1), granuloma (n = 1), keloid (n = 1), lipoma (n = 1), liposarcoma (n = 1), lymphoma (n = 1), malignant ulcer (n = 1), trauma (n = 1) and subcutaneous phycomycosis (n = 1). There were 72 (28.9%) cases without a documented clinical diagnosis. The majority (68.3%, 170 cases) of the cases or their biopsy specimens were referred from surgical units of both private and public hospitals around the country. Other referring units included medical outpatient (n = 9), medical inpatient (n = 4), orthopedic (n = 4), skin clinics (n = 3), cancer (n = 2), antenatal and gynecology (n = 1). Fifty-five cases had no referring unit recorded.

#### Distribution of mycetoma in Uganda

**Fig 2:**
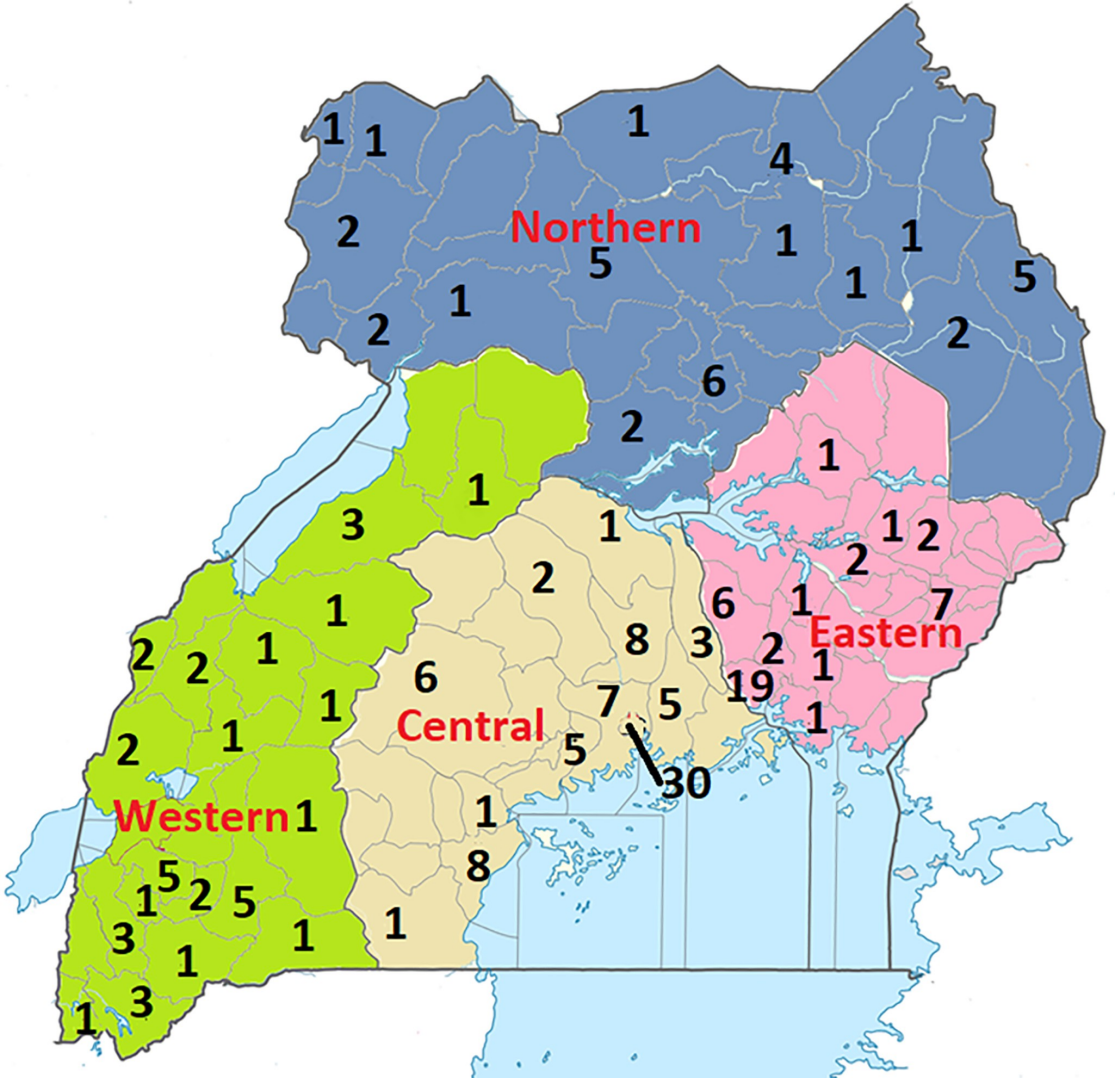
Distribution of mycetoma cases by district. There were 77 cases from the central, 43 cases from the East, 37 from the West and 35 cases from the North. There were also 57 cases without a record of the district of residence, and these were not included in this map. Districts with no cases were left blank. Map was created using the Microsoft Paint app in windows 10.

With regard to the tribe of the patients, majority of the cases were from the tribes of Baganda (34.1%, n = 85), Basoga (12.5% n = 31), Banyankole (9.2% n = 23), Acholi (6.8% n = 17) and Karamojong (4.0% n = 10). The rest of the tribes had less than 10 cases each, but the majority had one case each. Uganda is divided into 134 districts and the capital city of Kampala, which are grouped into four administrative regions, i.e., Northern, Western, Eastern and Central region. However, cases were registered from only 58/134 districts. Using data from the district of residence of the patients, we plotted the number of cases per district and region to determine the spatial distribution. The highest number of cases was recorded from Kampala district (n = 30) and Jinja district (n = 19). Other districts had less than 10 cases each over the seventy-year. The distribution of cases was almost even across the four regions (Fig 2).

**Fig 3:**
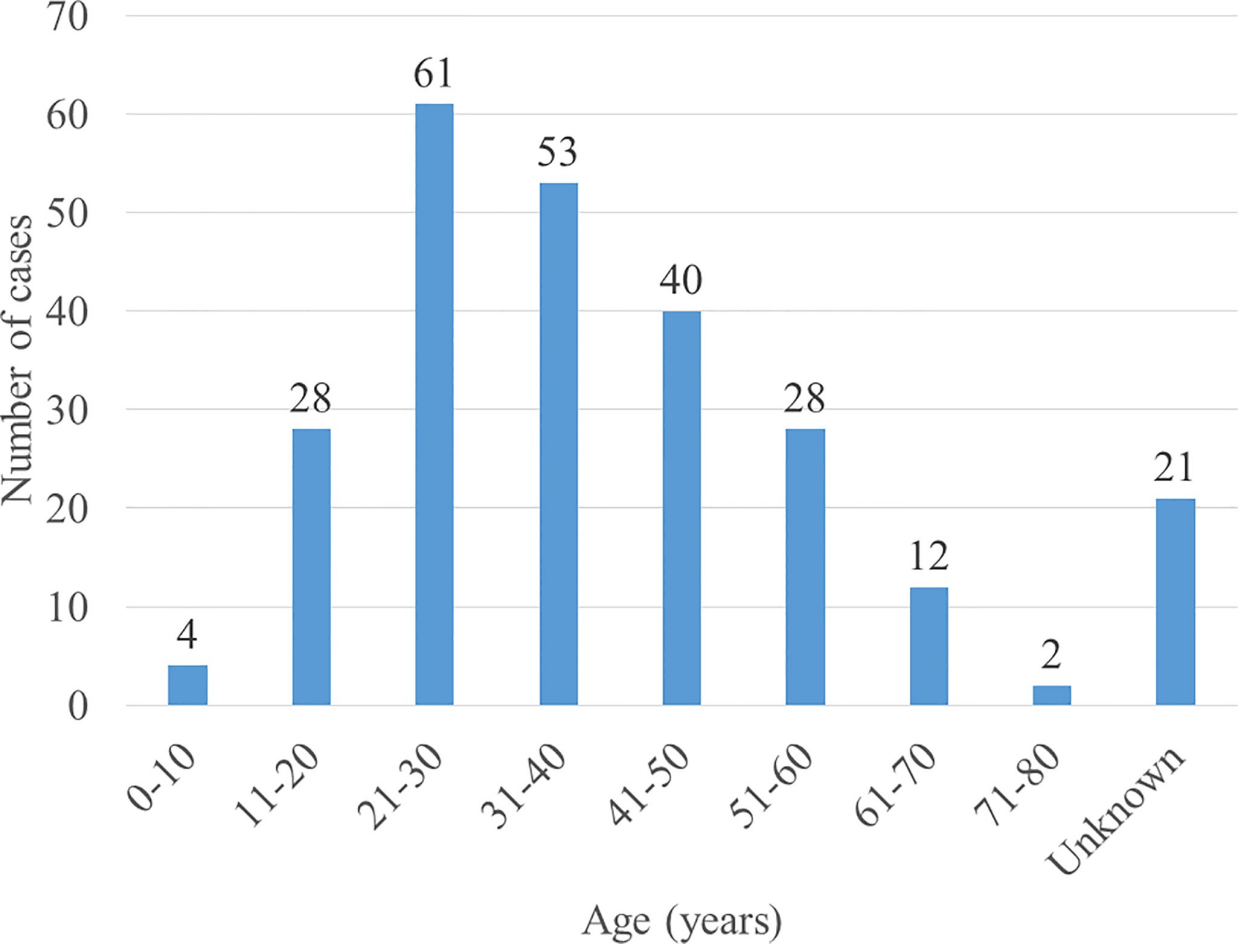
Age distribution of mycetoma patients. The age group most affected was 21–30 years.

Majority of the cases were males (56.6% [141/249]) with an overall median age for all cases being 37 years (IQR = 26–50, n = 228). Adults (≥ 18 years) comprised of 92.8% (231/249) of all cases. 21/231 (9%) adults had missing age and were recorded as “adult” in the reports. The age group most affected was 21–30 years (Fig 3) mostly caused by fungi (89%).

**Fig 4:**
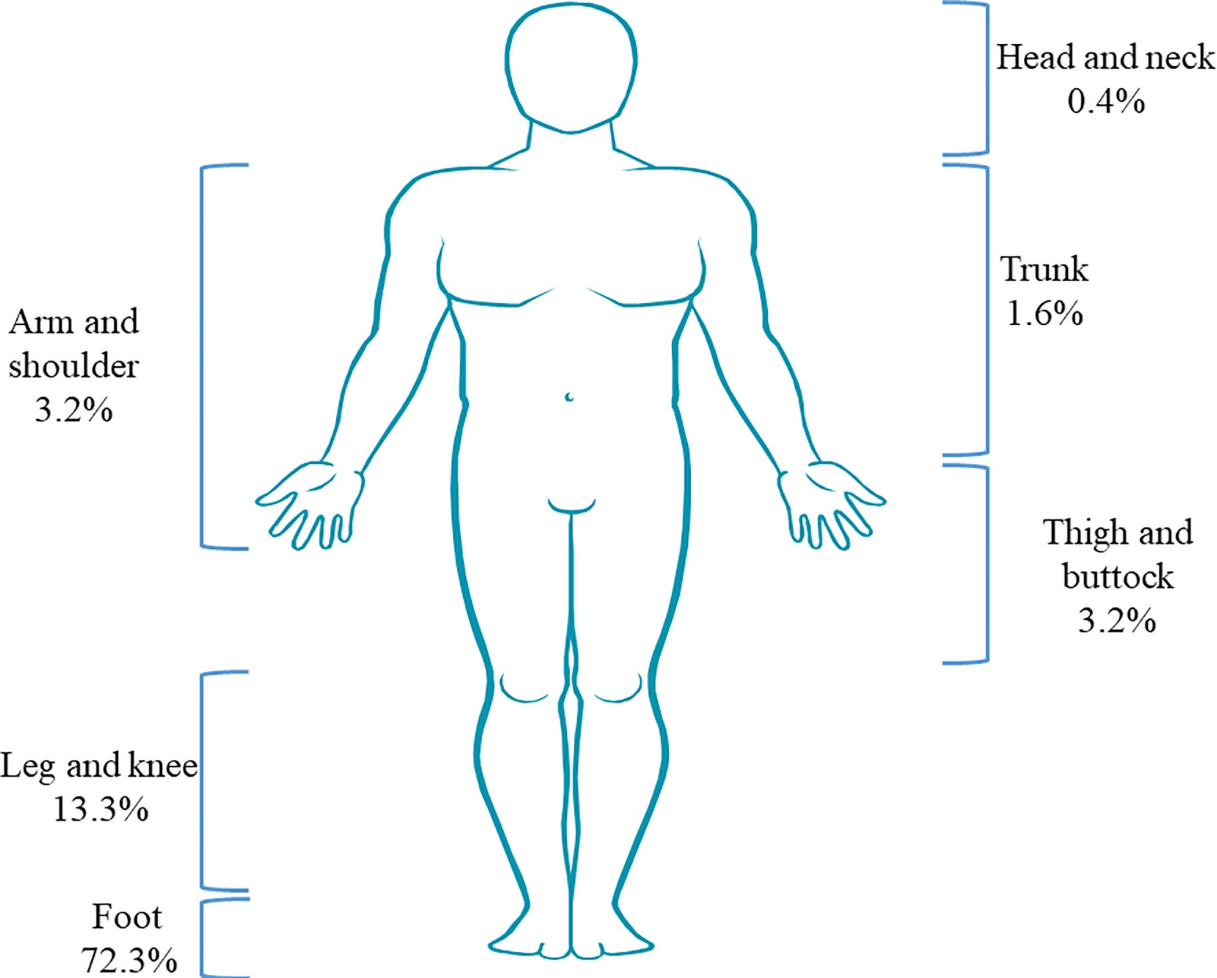
Mycetoma lesion site. The foot was the most affected site. For 6% (15) of the cases, the lesion site was unknown or not recorded.

With regard to the mycetoma lesion site, the foot was the most involved site (72.3%) followed by the leg and knee (13.3%), thigh and buttocks (3.2%), arm and shoulder (3.2%), trunk (1.6%) then head and neck (0.4%) (Fig 4). 26/249 cases had bone involvement documented while 15/26 had amputation of the limbs recorded (Table 1).

## Discussion

The results of this study indicate that mycetoma is an uncommon disease in Uganda, with an estimated 3,683 people living with the disease. In addition, data are scanty about mycetoma and its associated complications in Uganda, most likely due to non-reporting of the cases or inability to make a confirmatory diagnosis of the disease. Two hundred seventy-nine cases were identified over a period of 70 years (1950–2019). Thirty cases were from literature review while the 249 cases were from biopsy reports. However, there was a general and significant decline in the number of cases detected recently.

The cases were more during the 1960s to early 1980s; then they “disappeared” and became scanty. It’s unclear whether this is due to an actual reduction in the incidence or reduction in clinical suspicion or just poor health-seeking habits or due to the massive efflux of Ugandan physicians and scientists in the late 1970s due to political unrest in the country. We estimate that Makerere pathology laboratory captures about 10% of the total cases even though it is a national reference laboratory. Majority of the cases should be in the community undiagnosed. Besides, the level of clinical suspicion was low with a very large differential diagnosis based on clinical notes. The surgical unit had a higher index of suspicion compared to other departments.

The two published reports identified were both reported from Mulago National Referral Hospital, Kampala, where the department of Pathology for Makerere University is located. From the literature review, it was clear that both histology and radiology are useful tools for the diagnosis of mycetoma in resource-limited settings where PCR is costly and not widely available. Histology ideally gives a presumptive diagnosis based on tissue reaction and a definite diagnosis based on the presence of grains containing the causative agent. The causative agent can then be isolated in culture. In report 1, the diagnosis was made using histology and according to the authors, the grains were rare. In the absence of grains mycetoma can’t be diagnosed. It is possible that not all patients had grains. There could have been some false positives, especially that the authors were more interested in the radiological findings.

The literature review also identified *Nocardia* species as one of the major causes of mycetoma in Uganda while the biopsy reports identified only 12/249 cases of *Nocardia*. However, the biopsy reports showed fungi as the most common causative agents. The only challenge was that the biopsy reports did not identify the genus or species of the fungi, and we did not have any record of follow up cultures if any. Global estimates identify *Madurella mycetomatis* fungus as the most prevalent causative agent worldwide [5]. It’s possible that *M*. *mycetomatis* made up the most significant percentage of these unidentified fungi seen using PAS staining.

The spatial distribution of cases was almost even across the four regions. We initially anticipated that more cases would come from the Northern districts that border South Sudan where mycetoma is endemic, but this was not the case. The 30 cases identified in Kampala district could be explained by the ease of access of the Pathology laboratory in the same district and the proximity to the National Referral Hospital. However, the 19 cases identified from Jinja district in the Eastern region was a surprise. This may in fact, represent an area of clustered disease and high prevalence. It is also important to note that Eastern Uganda is highly endemic of *Tunga penetrans*, which may facilitate inoculation of agents of maduromycosis. Similarly, the tribes from the central and eastern region had the highest number of cases.

Similar to published literature [5,19], males and the age group of 21–30 were most affected by mycetoma in Uganda, and only 7% of all cases were children aged between 4 and 17 years. The foot was the most affected part of the body, followed by the leg. Ten per cent of the cases had bone involvement of which 58% underwent amputation.

Mycetoma is a Neglected Tropical Disease estimated to affect over 8,000 individuals globally [5]. Due to general lack of or inaccessibility to pathological services across the country, it is also possible that many patients were clinically diagnosed with mycetoma and were/are managed empirically on a combination of antifungal and antibacterial agents. Experience from Sudan has shown that establishing a centre of excellence for mycetoma improved active case findings, diagnosis and management of the disease [21]. Mycetoma Research Centre in Sudan alone cares for over 8,800 patients, a value slightly higher than the earlier global estimate of the burden of mycetoma [5]. Thus, there is an urgent need to update on the estimate of the global burden of mycetoma.

The study limitation includes the fact that cases were found by searching archives from a single hospital in only one city. There were missing data due to blank entries on the biopsy reports. Fungal pathogens were not identified to genus or species level. There was no data about the treatment of the cases. It is possible that the diagnosis for the two published reports was done in the reference Pathology laboratory where the 249 cases were diagnosed, which could lead to some cases being duplicated. However, this is the largest description of mycetoma in Uganda.

Despite the limitations to the study, we retrospectively gave a good overview of our current knowledge on the burden estimate for mycetoma in Uganda, highlighting that it is a rare disease and there is a big gap in data and epidemiological studies. Further studies are merited, including active community-based case findings.

## Supporting information

S1 Dataset Dataset for biopsy reviews. (XLSX)

S1 Checklist STROBE checklist for observational studies. (PDF)
